# Cigar package quantity and smoking behavior

**DOI:** 10.1186/s12889-019-7205-3

**Published:** 2019-07-03

**Authors:** Alexander Persoskie, Erin Keely O’Brien, Elisabeth A. Donaldson, Jennifer Pearson, Kelvin Choi, Annette Kaufman, Cassandra A. Stanton, Cristine D. Delnevo

**Affiliations:** 1 Office of Science, US Food and Drug Administration (FDA) Center for Tobacco Products, Building 71, Room G335, 10903 New Hampshire Avenue, Silver Spring, MD 20993 USA; 2 Division of Social and Behavioral Health/Health Administration and Policy, School of Community Health Services, University of Nevada, Reno, NV USA; 3 Department of Health, Behavior, and Society, Johns Hopkins Bloomberg School of Public Health, Baltimore, MD USA; 4 Division of Intramural Research, National Institute on Minority Health and Health Disparities, Bethesda, MD USA; 5 Tobacco Control Research Branch, Behavioral Research Program, Division of Cancer Control and Population Sciences, National Cancer Institute, Bethesda, MD USA; 6 Westat, Rockville, MD USA; 7 Rutgers School of Public Health, Center for Tobacco Studies, New Brunswick, NJ USA

**Keywords:** Pack quantity, Pack size, Package size, Packaging, Cigar, Tobacco packaging

## Abstract

**Background:**

Several jurisdictions in the US and abroad limit the minimum number of cigars that can be sold per package. Research has not evaluated whether small packages might result in cigar use initiation, or whether adding cigars to packages might result in purchasers smoking more cigars.

**Methods:**

Using nationally representative US adult data from Waves 1 and 2 of the Population Assessment of Tobacco and Health (PATH) Study, we assessed links between cigar package quantity (number of cigars in the package a person usually buys) and (1) price, and (2) cigar and cigarette use over time, for three cigar types: filtered cigars, cigarillos, and large cigars.

**Results:**

Smaller quantity packages (i.e., packages with fewer cigars) were cheaper per-pack than larger quantity packages but more expensive per-stick for all three cigar types. For filtered cigars, past-year starters tended to buy smaller quantity packages compared to longer-term users (geometric mean = 6.31 vs. 11.75, respectively; *b* = −.18, 95%CI: −.32, −.04). Also, those who bought smaller quantity packages of filtered cigars tended to smoke fewer cigars over time compared to those who bought larger quantity packages (*b* = 1.16, 95%CI: 0.45, 1.87). Neither of these associations was observed for cigarillos or large cigars. We also found little evidence that buying larger quantity packages predicted continuing to use cigars or using cigarettes.

**Conclusions:**

Although we found consistent associations between package quantity and price, we found few associations between package quantity and changes in cigar smoking behaviors over time, particularly for cigarillos and large cigars. Key limitations include our adult-only analyses and inability to determine the package quantity that cigar users initiated with. Future studies could examine whether package quantity plays a causal role in filtered cigar use initiation or consumption rates.

## Background

**Fig. 1:**
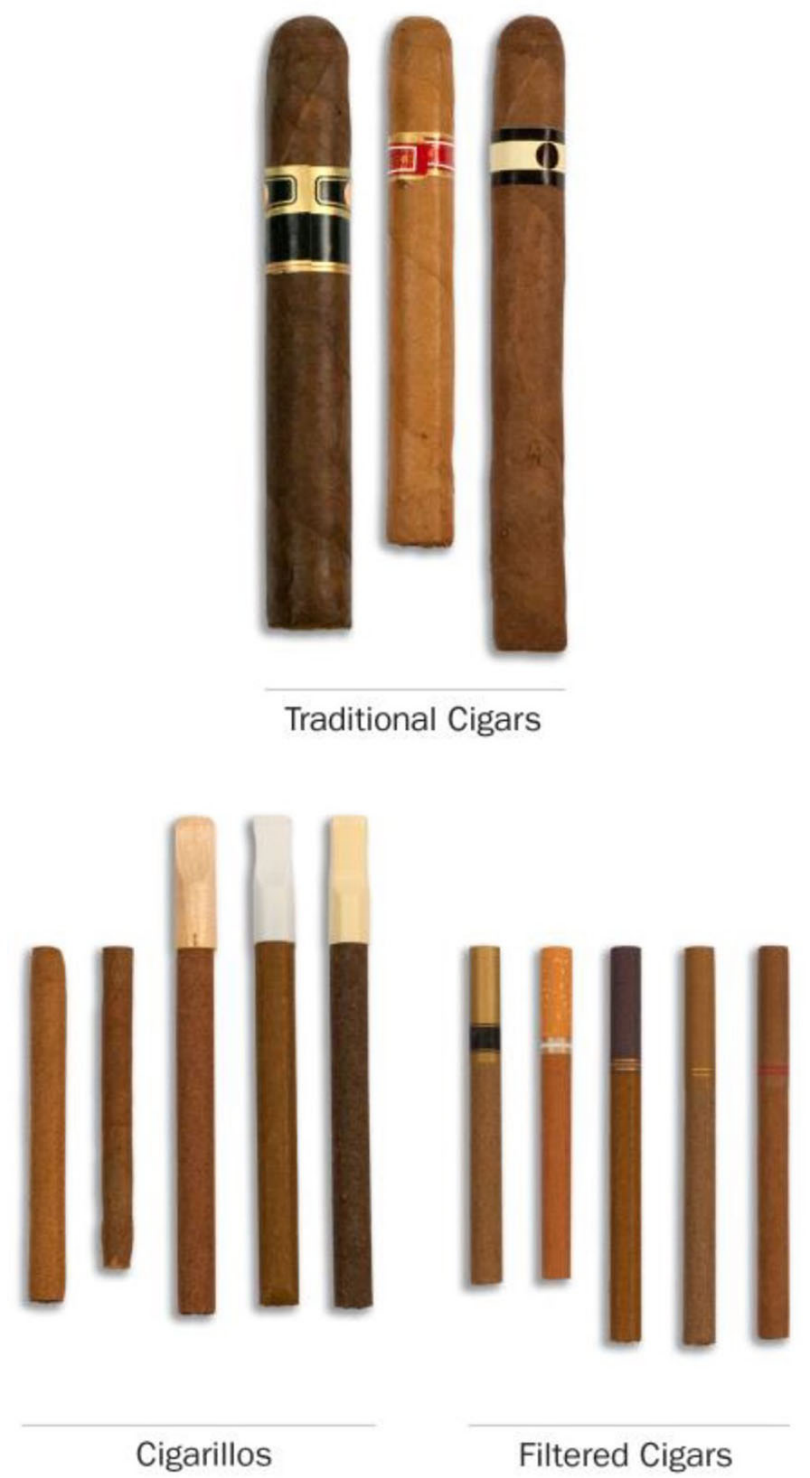
Images of cigar types, as presented to participants in the Population Assessment of Tobacco & Health (PATH) Study. Image credit: PATH Study. Note: The PATH Study referred to large cigars as “traditional cigars.” The PATH Study introduced the cigar types as follows at Wave 1: “The next questions are about traditional cigars, cigarillos, and filtered cigars. These products go by lots of different names, so please use these descriptions and photos to understand what they are. Traditional cigars contain tightly rolled tobacco that is wrapped in a tobacco leaf. Some common brands of cigars include Macanudo, Romeo y Julieta, and Arturo Fuente, but there are many others.” “Cigarillos and filtered cigars are smaller than traditional cigars. They are usually brown. Some are the same size as cigarettes, and some come with tips or filteres. Some common brands are Black & Mild, Swisher Sweets, Dutch Masters, Phillies Blunts, Prime Time, and Winchester.” To distinguish between cigarillos and filtered cigars, participants were asked if they had used the kinds of cigarillos or filtered cigars “with a plastic or wooden tip,” “with a filter (like a cigarette filter),” and “without a tip or filter” (“Choose all that apply”)

In 2016, an estimated 22.7 million people aged 12 or older in the US used a cigar in the past year, and 12.2 million used a cigar in the past month [1]. Cigars – defined as tobacco rolls wrapped in either tobacco leaf or another tobacco-containing substance [2] – can be roughly divided into three types: filtered, cigarillo, and large cigars. As shown in Fig. 1, filtered cigars have a filter and can be similar in size to cigarettes (“little”) or slightly larger, cigarillos are mid-sized and do not have a filter but sometimes have a plastic or wooden tip, and large cigars are thick and have no filter or tip. Filtered cigars are commonly sold in packages of 20 sticks, while cigarillos and large cigars are commonly sold in packages of five or fewer [3]. Within each cigar type, cigars vary in attributes like size, weight, characterizing flavor, and quantity sold per package [3]. Using cigars is associated with increased mortality risk from lung cancer, tobacco-related cancers, and all-causes [4], although the risks may differ based on product sub-type and characteristics (e.g., smoke pH, which affects ease of inhalation) [2].

Sales of each cigar type in the US have changed between 2008 and 2015. Little filtered cigar sales fell drastically following a 2009 federal tax increase, while sales of other cigar types increased, due in large part to manufacturers converting little cigars to larger sizes (i.e., based on weight) to take advantage of lower tax rates [5, 6]. Correspondingly, the proportion of convenience store cigar sales made up by 2- and 3-packs of cigarillos increased from 1% in 2008 to 40% in 2015, while the proportions of sales made up by 5-packs and single sticks decreased (from 43 to 22% and from 33 to 26%, respectively) [3]. It is unknown whether these or other shifts in cigar package quantities had implications for cigar use initiation or consumption rates.

Researchers and public health advocates argue that selling tobacco products in small quantity packages reduces barriers to trial and initiation because of the relatively low price compared to larger quantity packages [7, 8]. High prices can deter tobacco use, including among youth [9–12]. Studies of cigarette smoking in Australia and Ireland suggest that, before the enactment of minimum package quantity laws for cigarettes, youth smokers typically bought cigarettes in packs of 15 or 10, whereas adult smokers typically bought larger packs [8, 13]. Researchers argue that the availability of “loosies” (i.e., cigarettes sold individually) promotes smoking [14] and discourages cessation [15] by cuing urges to smoke and “allow[ing] for those with fewer resources to buy cigarettes without having to purchase a pack” [14]. Following tax-induced price increases for cigarettes, people may switch from cigarettes to lower-priced cigars rather than quitting [4, 5, 9].

Concerns about price sensitivity and cigar use initiation have led lawmakers and policy advocates in the US to seek prohibitions on small quantity, low-priced cigar packages. For example, Boston prohibits sales of cigars in packages of fewer than four unless the per-stick price is at least $2.50 [16], and three Minnesota cities have enacted similar laws [17]. These restrictions on cigar price and quantity have led to reduced availability of small quantities (singles and two- and three-packs) and increased prices of cheap cigars at local tobacco retailers [16, 17]. In 2013, New York City began requiring filtered cigars to be sold in packages of at least 20 sticks for a minimum of $10.50 per-package, and it required other cigars priced at $3 or less per-stick to be sold in packages of at least four [18]. After the passage of New York City’s 2013 law, there was a “proliferation of inexpensive 4-packs of cigars” (e.g., four cigars for $.99), causing a reduction in the average price of 4-packs [19]. In 2017, New York City enacted a new law that set the minimum price for a package of cigars at $8 for a single cigar and another $1.75 for each additional cigar per package [19]. US federal law does not restrict cigar package quantity but does restrict the minimum quantity for cigarettes (20 sticks) (21 CFR 1140.16).

It is also possible that cigar package quantity plays a role in current users’ consumption frequency. A study of tobacco industry research documents from the 1980s and 1990s found that some cigarette smokers preferred small packs to self-regulate their smoking and achieve or maintain a desired consumption rate – particularly among occasional smokers and those seeking to cut down or quit smoking [20]. Consequently, some companies expected that their overall sales volume could decrease if they made smaller pack quantities available [20]. A study in a convenience sample of US smokers found that approximately one-third would prefer to purchase their cigarettes in a 10-pack rather than a 20-pack, with most (70%) believing that the smaller pack would help limit their smoking, and two-thirds indicating that they would be willing to pay at least a 10% price premium to buy the 10-pack [21]. A study of cigarette smokers in Minnesota found that those who bought cigarettes by the carton were less likely to cut back on their smoking and attempt to quit smoking in the next year, compared to those who bought cigarettes by the pack [22]. However, research in Mexico found limited evidence that cigarette loosies are used as a tool for quitting or reducing consumption [23].

The purpose of this research is to examine associations among cigar package quantity, cigar price, and cigar smoking given the wide variation in cigar package quantities. Using data from the national, longitudinal Population Assessment of Tobacco and Health (PATH) Study, we assessed how cigar package quantity relates to price and cigar use over two time-points for filtered cigars, cigarillos, and large cigars. Each cigar type was examined separately because of potential differences in user characteristics, products, use behaviors (e.g., puffing, inhaling, use frequency), and how the products are sold [24–26]. We examined whether, compared to longer-term users, people who recently started using cigars purchased smaller quantity packages, and whether buying larger, rather than smaller, packages predicted increasing one’s cigar smoking over time, continuing to smoke each cigar type, and continuing or starting to smoke a different cigar type or cigarettes.

## Methods

### Data source

The PATH Study is an ongoing, nationally-representative, longitudinal cohort study of US adults and youth. The current paper reports on adult data (ages 18+) from Waves 1 and 2, collected approximately one year apart from September 2013–December 2014 and October 2014–October 2015, respectively. We excluded youth because the youth study did not administer key items used in the present analyses. The PATH Study used a stratified, address-based, area-probability sampling design at Wave 1 that oversampled adult tobacco users, African Americans, and young adults (18–24 years). Audio computer-assisted self-interviews were conducted in-person with 32,320 adults (Wave 1) and 28,362 adults (Wave 2) with an overall weighted response rate of 83.2%. Westat’s institutional review board approved the study. Further PATH Study details are published elsewhere [27] (10.3886/Series606).

### Measures

*Cigar types.* Participants reported whether they had ever used filtered cigars, cigarillos, and large cigars (referred to in the survey instrument as “traditional cigars”). For each cigar type, the questionnaire displayed a photo of example products, described their physical characteristics, and listed popular brands (see Fig. 1) [25]. Ever users reported whether they currently used the cigar type “every day,” “some days,” or “not at all,” and their frequency of using cigars as blunts (i.e., with the filler tobacco replaced with marijuana).

*Package quantity and price.* For each cigar type, users reported how they usually purchased the cigar type, including “in-person (such as at a store or cigar bar),” “from the internet,” “by telephone,” or “I do not buy my own [cigar type]”. Those purchasing in-person were asked whether they usually purchased the cigar type as a single stick or in a box or pack of multiple cigars. Those who purchased boxes or packs reported how many cigars came in the box or pack they usually purchased.

For each cigar type, participants reported how much they paid for the box, pack, or single cigar they usually purchased. We created variables for (1) the total price and (2) the per-stick price (i.e., total price divided by package quantity). We divided large cigars into two subcategories based on whether they cost more than $2.50 per-stick or less than or equal to $2.50 per-stick. We based this on minimum package quantity laws in Boston and New York City, which exempt cigars that are at least $2.50 and $3.00 per-stick, respectively.

*Cigars smoked in past 30-days.* For each cigar type, users reported the number of cigars they smoked on days when they used the cigar type in the past 30 days. Also, users who smoked a cigar type “some days” reported how many days they smoked the cigar type in the past 30 days. For users of each cigar type, we calculated the number of cigars of that type smoked in the past 30 days by multiplying the number of cigars the participant smoked per day by 30 (for “every day” users), or by the number of days smoked in the past 30 (for “some day” users).

*Cigarette use.* We defined cigarette smokers as those who reported currently smoking cigarettes “every day” or “some days”. We did not require a minimum level of lifetime use (e.g., ≥100 cigarettes), consistent with our definitions of current cigar use (see below), in order to include smokers who may have started recently.

### Analyses

Analyses included adult current users of each cigar type (filtered, cigarillo, and large), defined as those who currently used the cigar type “every day” or “some days.” Analyses of each cigar type were not mutually exclusive (i.e., participants could be users of multiple cigar types). Because of the low number of cigar users smoking large cigars that cost over $2.50 each, we excluded analyses of these large cigars. Also, we excluded participants who reported using cigars as blunts every time they smoked a cigar in the past 12 months, given that blunts do not meet this paper’s definition of a cigar (i.e., a tobacco roll wrapped in either tobacco leaf or another tobacco-containing substance) [2] and are likely to be used differently than cigars (e.g., because of differences in accesss to and legality of marijuana). Removing blunt-only users excluded 38 filtered cigar, 403 cigarillo, and 116 large cigar users from Wave 1; for Wave 2, the PATH Study interview automatically identified blunt-only users, who we then excluded. At each wave, analyses were also restricted to users of each cigar type who had data on package quantity for that cigar type (see Table 1 for *n*s).

**Table 1:** Package quantity means and frequencies for filtered cigars, cigarillos, and large cigars in PATH Study Waves 1 and 2

				Package quantity Weighted % (Unweighted n)				
Cigar type	Wave	N	Weighted geometric mean (SE) package quantity	Single	2–3 pack	4–5 pack	6–19 pack	20+ pack
Filtered	1	514	12.02 (1.05)	13.06 (82)	1.09 (7)	4.45 (21)	14.80 (78)	66.60 (326)
	2	428	9.12 (1.07)	20.08 (94)	2.95 (16)	4.27 (22)	14.74 (65)	57.96 (231)
Cigarillo	1	901	2.46 (1.05)	48.95 (458)	12.34 (124)	24.69 (205)	4.43 (43)	9.59 (71)
	2	731	2.24 (1.05)	50.28 (395)	15.85 (113)	21.92 (144)	4.60 (32)	7.35 (47)
Large	1	441	3.63 (1.07)	39.17 (184)	5.09 (28)	26.10 (110)	10.58 (42)	19.06 (77)
	2	210	2.95 (1.10)	41.06 (92)	12.27 (29)	22.30 (43)	12.08 (24)	12.28 (21)

*Note.* Reported sample sizes are unweighted. Weighted %s and unweighted n’s are based on untransformed package quantities. For calculating means and Standard Errors (SEs), package quantity was base-10 log-transformed; means and SEs were then back-transformed

We used linear and logistic regressions to examine package quantity associations with price and product use behavior. We conducted analyses separately for each cigar type using SAS 9.4 and SUDAAN 11.0. We weighted estimates to represent the US adult population and estimated standard errors and 95% confidence intervals (CIs) using the balanced repeated replication method [28] with Fay’s adjustment set to 0.3 to increase estimate stability [29]. Descriptive analyses used weights corresponding to the reported statistic’s wave. Except for analyses of associations between price and package quantity (which used Wave 1 data only), inferential analyses were longitudinal and used Wave 2 weights. Adjusted analyses controlled for sex (male, female), age, race/ethnicity (White, Black, Hispanic, other), education (less than high school or General Educational Development [GED], high school, some college or associate’s degree, bachelor’s degree, advanced degree), and income. Missing data on these variables were not imputed.

Package quantity and price distributions included several extreme outliers, were highly positively skewed, and did not meet normality assumptions. We Winsorized extreme outliers on price (two filtered cigar values at Wave 1) and package quantity (one filtered cigar value, three cigarillo values, and three large cigar values at Wave 1), replacing them with the next highest value in the distribution [30]. We base-10 log-transformed package quantity, price, and past 30-day smoking frequency variables. We report back-transformed results where noted [31].

## Results

### Descriptive analyses

Table 1 shows weighted package quantity distributions for each cigar type. At both waves, package quantity was lowest for cigarillos (geometric mean [*GM*] = 2 per package) and highest for filtered cigars (*GM* = 9–12 per package, across waves). Cigarillos were most commonly purchased as singles (49–50%) or 4–5 per package (22–25%). Large cigars were also most commonly purchased as singles (39–41%) followed by 4–5 per package (22–26%). Filtered cigars were most commonly purchased in packages of 20 or more (58–67%), but singles (13–20%) and packages of 6–19 (15%) were also reported.

### Package quantity and price

Table 2 shows the weighted geometric mean prices paid for various cigar package quantities at Wave 1, both per-pack and per-cigar. Weighted simple linear regressions found that, for all cigar types, larger packages tended to be more expensive *per-pack* than smaller packages (*b*_FILTERED_ = 0.23, 95%CI: 0.17,0.29; *b*_CIGARILLO_ = 0.54, 95%CI: 0.46,0.61; *b*_LARGE_ = 0.52, 95%CI: 0.43,0.62) but less expensive *per-stick* (*b*_FILTERED_ = − 0.77, 95%CI: − 0.83,-0.71; *b*_CIGARILLO_ = − 0.47, 95%CI: − 0.55,-0.39; *b*_LARGE_ = − 0.50, 95%CI: − 0.60,-0.40).

**Table 2:** Weighted geometric mean prices of cigars (per pack and per cigar) by package quantity in the PATH Study at Wave 1, in US dollars

		Package quantity				
Cigar type	Per pack or per cigar	Single	2–3 pack	4–5 pack	6–19 pack	20+ pack
Filtered	Per Pack	1.48	1.45	5.50	5.89	3.02
	Per Cigar	1.48	0.69	1.15	0.56	0.14
Cigarillo	Per Pack	1.17	1.48	4.37	5.62	3.80
	Per Cigar	1.17	0.62	0.91	0.65	0.17
Large	Per Pack	1.26	1.70	4.37	6.61	4.90
	Per Cigar	1.26	0.69	0.89	0.78	0.20

*Note.* Prices were base-10 log-transformed for calculation of means, which were then back-transformed to geometric means

### Package quantity and past-year starting

Weighted linear regressions tested whether past-year starters at Wave 2 (i.e., people who were users of a cigar type at Wave 2 but not at Wave 1) bought smaller packages compared to longer-term users (i.e., people who were users of the cigar type at both waves; see Table 3). For filtered cigars, past-year starters bought smaller packages (*GM* = 6.31) than did longer-term users (*GM* = 11.75), an association that was significant in unadjusted and adjusted models. For large cigars, the same pattern emerged but did not reach statistical significance in either model (*GM* = 2.40 vs. *GM* = 3.31, respectively), and no difference emerged for cigarillos (*GM* = 2.29 and *GM* = 2.24, respectively).

**Table 3:** Wave 2 cigar use status (past year starter vs. longer-term user) predicting Wave 2 package quantity in the PATH Study

Cigar type	Wave 2 use status	Wave 2 package quantity Geometric mean (95% CI)	Bivariate *b* (95% CI)	Adjusted *b* (95% CI)^a^
Filtered	Past-Year Starter	6.31 (5.02, 7.94)	**−.27(−.40, −.15)**	**−.18(−.32,-.04)**
	Longer-Term User	11.75 (10.00, 13.80)		
Cigarillo	Past-Year Starter	2.29 (1.95, 2.75)	.01(−.07, .10)	.01(−.07, .09)
	Longer-Term User	2.24 (2.00, 2.46)		
Large	Past-Year Starter	2.40 (1.78, 3.31)	−.13(−.29, .03)	−.12(−.26, .03)
	Longer-Term User	3.31 (2.63, 4.07)		

*Note*. *b* = unstandardized beta coefficients from linear regressions of being a Wave 2 new user (vs. longer-term user) predicting Wave 2 package quantity. Past year starters were current users of the cigar type at Wave 2 but not Wave 1. Longer-term users were users of the cigar type at Wave 1 and Wave 2. Package quantity was base-10 log-transformed for analysis. Reported means and confidence intervals (CIs) are back-transformed. *n*’s for the bivariate analyses were 428 (filtered cigars), 731 (cigarillos), and 210 (large cigars). Bold typeface indicates *p* < .05

^a^Adjusted for sex, age, race/ethnicity, education, and income

### Package quantity and changes in past 30-day cigar use frequency

Among people who were users of each cigar type at both waves, weighted linear regressions assessed whether Wave 1 package quantity predicted Wave 2 number of cigars smoked in the past 30 days, adjusting for Wave 1 number of cigars smoked in the past 30 days (see Table 4). No significant effects were observed for cigarillos or large cigars. However, for filtered cigars, buying a larger package at Wave 1 predicted smoking a greater number of cigars in the past 30 days at Wave 2, adjusting for Wave 1 past 30-day filtered cigar use.

**Table 4:** Wave 1 package quantity predicting change in cigars smoked in the past-30 days (number of days in past-30 days smoked x number of cigars smoked per day) from Wave 1 to Wave 2 in the PATH Study

	Change in log10 (number of cigars smoked in past-30 days), Wave 1 to Wave 2	
Cigar type	*b* (95% CI)	Adjusted *b* (95% CI)
Filtered	**1.28** **(0.66, 1.90)**	**1.16** **(0.45, 1.87)**
Cigarillo	−0.14 (−0.58, 0.30)	−0.18 (−0.69, 0.34)
Large	0.02 (−0.54, 0.59)	−0.27 (−1.14, 0.60)

*Note*. *b* = unstandardized regression coefficient from linear regression, with Wave 1 package quantity predicting Wave 2 number of cigars smoked in past-30 days (adjusting for Wave 1 number of cigars smoked in past-30 days). *n*’s for these analyses were 148 (filtered cigars), 182 (cigarillos), and 77 (large cigars). Adjusted analyses also controlled for sex, age, race/ethnicity, education, and income. Number of cigars smoked in past-30 days was estimated by multiplying number of days of the last 30 smoked by number of cigars they smoked per day. Both the outcome and predictor variables were base-10 log-transformed for all analyses. Coefficients were not back-transformed. Participants were excluded from any analyses for which they lacked data on any predictor or outcome variable. Bold typeface indicates *p* < .05

Although package quantity for filtered cigars was positively associated with changes in cigar smoking over time, this effect did not appear to be driven by packages with fewer than 20 cigars vs. those with 20 or more cigars, based on supplemental analyses. Specifically, we examined changes in past 30-day filtered cigar smoking frequency between Waves 1 and 2, stratified by whether people bought packages with fewer than 20 cigars compared to 20 or more cigars at Wave 1. Although people who purchased filtered cigars in packages of 20 or more at Wave 1 smoked many more cigars at Wave 2 than those who bought smaller packages, they did not *increase* their cigar smoking compared to Wave 1 purchasers of filtered cigars in smaller packages *(data not shown)*.

### Package quantity and changes in cigar and cigarette use status

Weighted logistic regressions tested whether, among Wave 1 users of each cigar type, buying larger packages at Wave 1 predicted continuing to use the cigar type at Wave 2 (see Table 5). Buying large cigars in larger quantity packages at Wave 1 predicted still being a large cigar user at Wave 2: Wave 1 users who continued to use at Wave 2 had purchased an average (*GM*) of 4.57 cigars per package at Wave 1, while those who were no longer users at Wave 2 had purchased an average (*GM*) of 3.16 cigars per package at Wave 1. However, after adjusting for demographics, this effect was not significant. There was a similar but non-significant pattern for filtered cigars (i.e., *GM*_USERS_ = 14.13; *GM*_NON-USERS_ = 11.22) but not for cigarillos (*GM*_USERS_ = 2.51; *GM*_NON-USERS_ = 2.57).

**Table 5:** Wave 1 package quantity predicting Wave 2 use of the same cigar type, use of any other cigar type, and cigarette use in the PATH Study

Cigar type	Wave 2 Use of Same Cigar Type	Wave 1 Package Quantity Geometric Mean (95% CI)	*OR* (95%CI)^a^	*AOR* (95%CI)^e^
Filtered	Non-User	11.22 (9.55, 12.88)	1.54 (0.98, 2.42)	1.26 (0.79, 2.00)
	User	14.13 (12.02, 16.22)		
Cigarillo	Non-User	2.57 (2.19, 3.02)	0.97 (0.64, 1.47)	0.98 (0.59, 1.64)
	User	2.51 (2.24, 2.88)		
Large	Non-User	3.16 (2.63, 3.80)	**1.77 (1.12, 2.82)**	1.39 (0.78, 2.47)
	User	4.57 (3.63, 5.89)		
Cigar Type	Wave 2 Use of Any Other Cigar Type^c^	Wave 1 Package Quantity Geometric Mean (95% CI)	OR (95% CI)^b^	AOR (95% CI)^e^
Filtered	Non-User	13.49 (11.75, 15.49)	0.73 (0.41, 1.28)	1.00 (0.53, 1.92)
	User	10.47 (8.51, 12.88)		
Cigarillo	Non-User	2.14 (1.91, 2.40)	**1.75 (1.15, 2.67)**	1.43 (0.86, 2.39)
	User	3.16 (2.69, 3.72)		
Large	Non-User	3.31 (2.57, 4.27)	1.33 (0.78, 2.27)	1.33 (0.70, 2.52)
	User	3.71 (3.16, 4.37)		
Cigar Type	Wave 2 Cigarette Use	Wave 1 Package Quantity Geometric Mean (95% CI)	OR (95% CI)^d^	AOR (95% CI)^e^
Filtered	Non-User	11.75 (9.55, 14.13)	1.11 (0.65, 1.89)	1.10 (0.50, 2.45)
	User	12.88 (11.22, 14.45)		
Cigarillo	Non-User	2.75 (2.34, 3.31)	0.73 (0.49, 1.07)	0.78 (0.47, 1.30)
	User	2.29 (2.04, 2.57)		
Large	Non-User	4.36 (3.55, 5.25)	0.65 (0.37, 1.13)	1.05 (0.45, 2.41)
	User	3.09 (2.57, 3.80)		

Note: Wave 1 package quantity was base-10 log-transformed for all analyses. Reported means and 95% CIs were back-transformed. *n*’s for the bivariate analyses of Wave 2 Use of Same Cigar Type were 440 (filtered cigars), 662 (cigarillos), and 328 (large cigars). *n*’s for the bivariate analyses of Wave 2 Use of Any Other Cigar Type were 441 (filtered cigars), 736 (cigarillos), and 352 (large cigars). *n*’s for the bivariate analyses of Wave 2 Cigarette Use were 441 (filtered cigars), 735 (cigarillos), and 352 (large cigars). In analyses of Wave 2 cigarette use, the *n*’s by Wave 2 cigarette smoking status were: filtered cigars (n_NON-SMOKER_ = 109; n_SMOKER_ = 332), cigarillos (n_NON-SMOKER_ = 240; n_SMOKER_ = 495), and large cigars (n_NON-SMOKER_ = 119; n_SMOKER_ = 233). Bold typeface indicates *p* < .05

^a^Odds ratio from using Wave 1 package quantity to predict Wave 2 use status

^b^Odds ratio from using Wave 1 package quantity to predict Wave 2 use status of any other cigar type, adjusting for use status at Wave 1

^c^Including large cigars > $2.50 per stick

^d^Odds ratio from using Wave 1 package quantity to predict Wave 2 cigarette smoking status, adjusting for smoking status at Wave 1

^e^Adjusted for sex, age, race/ethnicity, education, and income

Weighted logistic regressions tested whether buying larger packages at Wave 1 predicted using any other cigar type at Wave 2, adjusting for Wave 1 use of other cigar types. As shown in Table 5, Wave 1 cigarillo users who bought larger packages were more likely than those who bought smaller packages to start or continue using at least one additional cigar type at Wave 2. Those who did not use at least one additional cigar type bought an average (*GM*) of 2.14 cigarillos per package at Wave 1, while those who used at least one additional cigar type bought an average (*GM)* of 3.16 cigarillos per package at Wave 1. This effect was not significant after adjusting for demographics, and no effects emerged for filtered cigars or large cigars.

Weighted logistic regressions examined whether buying each cigar type in a larger package at Wave 1 predicted smoking cigarettes at Wave 2, adjusting for Wave 1 cigarette smoking status. No significant effects emerged (Table 5).

## Discussion

This study provides the first longitudinal analysis of nationally-representative US data to investigate associations among cigar package quantity, price, and cigar smoking. Given public health efforts to limit the minimum number of cigars that can be sold per package [7, 16–18] and the US Food and Drug Administration’s (FDA’s) authority to regulate package quantity as a cigar characteristic, results of this study can inform policy options. Although we found associations between package quantity and price, we found few associations between package quantity and cigar smoking behaviors.

### Package quantity, total price, and past-year starters

For each cigar type, as package quantity decreased, so did package price. This lends support to public health concerns that small packages are relatively inexpensive and thus may lower barriers to trial and initiation by non-users [7]. However, this finding also accords with internal tobacco company documents describing small packages as offering a low cost way for current users to try and potentially switch to new brands, particularly in markets with rising prices and among people who are younger and poorer [20].

We also found evidence that past-year starters of filtered cigars bought smaller packages compared to longer-term users. This fits the hypothesis that small packages encourage product trial and initiation because of their lower out-of-pocket costs [7, 8], but it also fits other explanations. For instance, past-year starters may consume fewer cigars and prefer small packages for that reason [20], and they may be more likely than longer-term users to sample different product varieties (in small packages). Quasi-experimental studies and different types of analyses (e.g., propensity score matching) may help determine whether the elimination of small packages might reduce filtered cigar initiation. For cigarillos and large cigars, average package quantities were low among past-year starters and longer-term users alike (2–3 per package), with no evidence that past-year starters preferred small packages more than longer-term users.

### Package quantity, per-stick price, and subsequent consumption

For all cigar types, as package quantity increased, the per-stick price of cigars decreased. There is little discussion of per-stick price in peer-reviewed literature; in contrast, tobacco companies appear to have considered it at length [20]. Analysis of tobacco industry documents suggests that cigarette companies have marketed large packages to reduce per-unit prices, to match the consumption rates of heavier users, to match the preferences of value-focused consumers, and sometimes to discourage people from quitting or switching to lower-priced competitor brands when prices increased or became more salient (e.g., following excise tax increases) [20].

For filtered cigars but not the other two cigar types, we found an association between cigar package quantity and subsequent cigar use. Among people who smoked filtered cigars at both waves of the study, buying smaller packages at Wave 1 predicted lower past 30-day cigar smoking frequency one year later at Wave 2. Of note, this analysis adjusted for past 30-day cigar smoking frequency at Wave 1, which reduces but does not eliminate the possibility that this finding merely reflects a preference for larger packages among people who smoke more cigars. Given that our study was observational, it does not provide evidence that current filtered cigar users’ consumption would increase if small package quantities were eliminated. However, our finding fits with prior studies suggesting that some people prefer to buy “vice” goods in small packages as a way to ration their product use [20, 21, 32]. Also, this effect was not driven by differences between packages with fewer than 20 filtered cigars compared to those with 20 or more cigars. The link between filtered cigar package quantity and changes in past 30-day cigar smoking remains an interesting area for future research.

We found little evidence that purchasing larger packages predicted continued cigar use one year later. In unadjusted analyses, large cigar users who purchased larger packages at Wave 1 were more likely to still use large cigars at Wave 2, and cigarillo users purchasing larger packages at Wave 1 were more likely to start or continue smoking at least one other type of cigar at Wave 2. However, neither effect remained statistically significant after adjusting for race/ethnicity, age, education, income, and sex. This suggests the possibility that cigar smokers with certain sociodemographic characteristics may be more likely to continue using cigars and also more likely to purchase larger package quantities. Future research could examine whether cigar companies target small or large package quantities based on sociodemographic factors, as has been observed for the marketing of non-standard pack quantities for cigarettes [20]. Indeed, one study of California tobacco retailers found that, with $1, consumers could buy larger packages of cigarillos in stores in poorer neighborhoods than in richer neighborhoods [33]. Two other retailer studies in Washington, DC [34] and California [35] suggested that it was possible to buy a cigarillo for a lower price in neighborhoods with higher percentages of African Americans and young people, but these studies only examined the per-stick price of the lowest priced pack for two brands. Socioeconomic disparities in the availability of single cigars appeared to remain after Boston implemented price and quantity regulations [16]. No significant associations emerged between cigar package quantity purchased at Wave 1 and cigarette smoking status at Wave 2, even though studies indicate that filtered cigars resemble and serve as economic substitutes for cigarettes [5, 9].

### Limitations

Some participants may have misclassified their cigar type. In particular, although the survey instrument presented photos and descriptions of each cigar type (see Fig. 1), some users may have misclassified cigarillos as filtered cigars, as suggested by the unexpectedly large number of filtered cigar users who reported that they were blunt-only users (*n* = 38 at Wave 1).

Our analyses of past-year starters were limited in two key ways: first, we did not exclude people if they had been users of the cigar type prior to Wave 1, meaning that some of our past-year starters may have been re-starters. Second, the purchases of past-year starters may not reflect the first few purchases that people make when they initially buy cigars.

Other limitations also warrant noting. This study could not assess whether package quantity played a causal role in cigar initiation or continued use. Our analyses also did not include youth, a primary group of concern among minimum package quantity law proponents. Our analyses only considered one aspect of cigars – package quantity – and did not consider other characteristics that may affect appeal, such as flavors. If flavors are associated with package quantity (e.g., if cigars sold in smaller packages are more likely to have characterizing flavors), this could have potentially confounded the associations we observed between filtered cigar package quantity and smoking behavior. However, we did not have a reason to expect an association between filtered cigar package quantity and flavors, and such associations would not explain our null findings for the other cigar types. Caution is warranted when comparing our results to other studies given that we did not distinguish between filtered cigars varying in length, weight, or other characteristics, whereas other studies separately examine *little* cigars and large filtered cigars [5]. We also did not consider whether variation in price-per-stick was associated with the weight of tobacco contained in cigarillos or large cigars. Our analyses provided a high-level overview of US adult cigar consumers rather than drilling down into specific consumer segments. Small sample sizes limited our ability to detect statistical associations and to stratify analyses by factors of interest such as demographic characteristics and blunt use frequency. Given the breadth of our analyses, we could not examine the potential role of blunt use; thus, we excluded people who reported using cigars as blunts only. Also, we could not fully address the role that income and other demographic characteristics may play in the purchase of small and large package quantities, although we did adjust our analyses for these characteristics.

## Conclusion

In our study of US adults, we found clear links between package quantity and the prices paid for three cigar types: smaller packages had lower overall prices but higher per-stick prices. Also, we found that past-year starters of filtered cigars bought smaller packages than did longer-term users, whereas we did not observe this pattern for users of cigarillos or large cigars. Buying filtered cigars in smaller rather than larger packages also predicted lower consumption frequency over time. To our knowledge, this study represents the first assessment of how cigar package quantity relates to cigar use, which may inform consideration of minimum cigar package quantity laws. We found few associations between cigar package quantity and use behavior and hope to encourage further empirical research in this understudied area of tobacco regulatory science.

## Data Availability

The datasets analyzed here are available upon request to the National Addiction & HIV Data Archive Program (NAHDAP) at doi:10.3886/ICPSR36231.v14 and the following link: https://www.icpsr.umich.edu/icpsrweb/NAHDAP/studies/36231.

## Abbreviations

FDA: United States Food and Drug Administration
GED: General Educational Development
GM: Geometric mean
PATH: Population Assessment of Tobacco and Health
SE: Standard Error
US: United States
